# Revealing the Specific Regulations of Brassinolide on Tomato Fruit Chilling Injury by Integrated Multi-Omics

**DOI:** 10.3389/fnut.2021.769715

**Published:** 2021-12-03

**Authors:** Chunmei Bai, Yanyan Zheng, Christopher B. Watkins, Anzhen Fu, Lili Ma, HongWu Gao, Shuzhi Yuan, Shufang Zheng, Lipu Gao, Qing Wang, Demei Meng, Jinhua Zuo

**Affiliations:** ^1^Key Laboratory of Vegetable Post-harvest Processing, Ministry of Agriculture, Beijing Vegetable Research Center, Institute of Agri-Food Processing and Nutrition, Beijing Academy of Agriculture and Forestry Sciences, Beijing, China; ^2^Beijing Key Laboratory of Fruits and Vegetable Storage and Processing, Beijing Vegetable Research Center, Institute of Agri-Food Processing and Nutrition, Beijing Academy of Agriculture and Forestry Sciences, Beijing, China; ^3^Key Laboratory of Biology and Genetic Improvement of Horticultural Crops (North China) of Ministry of Agriculture, Beijing Vegetable Research Center, Beijing Academy of Agriculture and Forestry Sciences, Beijing, China; ^4^Key Laboratory of Urban Agriculture (North) of Ministry of Agriculture, Beijing Vegetable Research Center, Beijing Academy of Agriculture and Forestry Sciences, Beijing, China; ^5^State Key Laboratory of Food Nutrition and Safety, College of Food Science and Engineering, Tianjin University of Science & Technology, Tianjin, China; ^6^School of Integrative Plant Science, Horticulture Section, College of Agriculture and Life Science, Cornell University, Ithaca, NY, United States

**Keywords:** brassinolide (BR), *Solanum lycopersicum*, transcriptome, metabolome, proteome

## Abstract

Tomato fruit is susceptible to chilling injury (CI) when stored at low temperatures, limiting its storage potential, and resulting in economic loss if inappropriate temperatures are used. Brassinolide (BR) is a plant growth regulator that is known to decrease the susceptibility of fruit to CI. In this study, transcriptome, metabolome, and proteome analysis revealed the regulation mechanism of BR treatment in alleviating tomato fruit CI. The results showed that the differentially expressed metabolites mainly included amino acids, organic acids, carbohydrates, and lipids. Differentially expressed genes (DEGs) were involved in plant cold stress response (*HSFA3, SHSP*, and *TPR*), fruit redox process (*POD, PAL*, and *LOX*), related to the fruit texture (*CESA*, β*-Gal*, and *PAE*), plant hormone signal transduction (*ACS3, ARF*, and *ERF*,), transcription factors (*TCP, bHLH, GATA*). Moreover, differentially expressed proteins were associated with fruit texture (CESA, PE, PL, and CHI), plant oxidation processes (LOX, GPX, CAT, and POD), plant cold stress response (HSF, HSP20, HSP70, and HSP90B), plant hormone signal transduction (BSK1 and JAR1) and transcription factors (WRKY and MYB). Our study showed that BR alleviates CI symptoms of tomato fruit by regulating LOX in the α-linolenic acid metabolism pathway, enhancing jasmonic acid-CoA (JA-CoA) synthesis, inhibiting cell wall and membrane lipid damage. The results provided a theoretical basis for further study on the CI mechanism of tomato fruit.

## Introduction

The tomato is a highly valuable vegetable that is widely cultivated in China and around the world (1, 2). Tomato (*Solanum lycopersicum*) fruits play a vital role in terms of both their nutritional value and their economic value (3). In order to delay the ripening process and extend the shelf life of harvested fruits, a non-freezing low-temperature treatment called cold storage or low-temperature storage is often used (4). Low-temperature storage is one of the most effective technologies for controlling the quality of fruits and vegetables after harvest (5). However, as a subtropical crop, tomato fruit, like other tropical and subtropical fruits, is sensitive to low temperatures and susceptible to chilling injury (CI) and has been widely used as a model for studying cold-induced responses (6, 7).

Chilling injury is a type of physiological defect that frequently occurs in the tissues of susceptible plants when they are exposed to low temperatures but not frozen (8, 9). The tomato, as a subtropical fruit, is susceptible to cold stress (7). CI manifests as pitting and lesions in the fruit, as well as irregular ripening (10). The severity of CI in tomato fruit is determined by internal factors, such as the cultivar and growing conditions, and external factors, such as the storage temperature and time (11). However, CI usually occurs when the fruit is stored for 6–8 days at temperatures of 5°C or less (12). Treatments with jasmonic acid, gibberellin, and oxalic acid, as well as atmosphere and humidity packaging, have been reported to reduce the development in the CI of tomato fruit (13–16).

Brassinolide (BR), a polyhydroxyl steroid, is a naturally occurring plant-growth regulator (17, 18) that affects a range of physiological processes in plants (19–21). BR is considered to be the most widely used plant hormone in post-harvest storage and the preservation of fruits and vegetables (22, 23). Several studies have shown that treatments with BR can delay the development of CI symptoms in cucumbers, green peppers, and bamboo shoots (17, 24, 25). BR can also accelerate the change in tomato fruit from the mature green to full red stages and can increase the contents of ascorbic acid and lycopene (19).

In this study, the regulatory role of BR treatment in alleviating tomato fruit CI was studied through combined metabolomic, transcriptomic, and proteomic analysis, in order to provide a new perspective on studying the molecular regulatory mechanisms for alleviating tomato fruit CI.

## Materials and Methods

### Sample Preparation

Tomato fruit was acquired at the green ripe stage from a glasshouse in Beijing Yanqing Dongxiaoying village (Yanqing District, Beijing, China) and transported directly to a laboratory. Roughly 200 tomato fruits of similar sizes and ripeness, excluding those exhibiting wounds or decay, were separated evenly into two portions. One group was submerged in 0.75 μmol/L BR solution for 10 min, and the other, in pure water for 10 min as a control. After air drying, both the control and treated groups were stored in baskets at 0 ± 1°C with 85–90% relative humidity. After cold storage, CI symptoms appeared in both groups; however, the CI symptoms were less apparent in the BR-treated group. During storage at 0 ± 1°C, the pericarp of the tomato fruit was sampled at 0 and 8 days. The samples at 0 d were set as the control group (CK). Three replicate samples of three fruits were collected, with pericarp tissues taken and frozen in liquefied nitrogen. The tissues were kept at −80°C for subsequent analysis.

### CI Index

The CI index was determined as described in a previously published article (16).

### RNA Extraction and Detection and Differential Expression Analysis

RNA degradation and contamination were monitored on 1% agarose gels. The RNA purity was checked using a NanoPhotometer® spectrophotometer (IMPLEN, CA, USA). The RNA integrity was assessed using the 2100 Bioanalyzer RNA Nano 6000 Assay Kit (Agilent Technologies, CA, USA). Differential expression analysis was based on former studies(26–28).

### GO and KEGG Enrichment Analysis

Gene ontology (GO) and Kyoto Encyclopedia of Genes and Genomes (KEGG) pathway enrichment analyses of differentially expressed genes were performed using the clusterProfiler (3.4.4) software (29, 30).

### Extraction and Identification of Metabolites

For each replicate, 100 mg of tissue was powdered in liquid nitrogen and placed in a fresh Eppendorf tube. Next, 500 u-L of 80% methanol (containing 0.1% formic acid), was added to the tube, and the mixture was vortexed, put on ice, and left to stand for 5 min. The tubes were centrifuged at 15,000 rpm for 10 min at 4°C, and the supernatant liquid was diluted to the final concentration (including 53% methanol) with liquid chromatography-mass spectrometry (LC-MS) grade water and then centrifuged at 15,000 × g at 4°C for 20 min. The supernatant liquid was used for analysis by LC-MS.

In accordance with the Novogene database, multi-reaction monitoring mode (MRM) was used to analyze the experimental samples. The compounds were quantified, and a qualitative analysis was conducted. SCIEX OS version 1.4 (SCIEX, USA) was used to open the off-machine mass spectrum file, and the chromatographic peaks were integrated and corrected according to a set minimum peak height of 500, a signal-to-noise ratio of 5, smooth points of 1, and other information. The chromatographic peaks were screened. Finally, the integral data of all the chromatographic peaks were derived to obtain the qualitative and quantitative metabolite results.

### Extraction, Identification, and Functional Analysis of Protein

The method for the extraction of protein from the tomato peel was based on previous research (31–35). The Proteome Discoverer 2.4 software (Guangzhou guangdian International Trade Co., LTD, Guangdong, China) was used to search and identify the differential proteins, and a *t*-test was used to analyze the quantitative protein results (36). Interproscan was used for GO functional annotation, and KEGG was used for the functional protein pathway analysis of the identified proteins (37, 38).

### Statistical Analysis

A one-way ANOVA was used for data comparison, and the least significant difference (LSD) test at *P* = 0.05 was used for the comparison of means. The figures were drawn using Origin 8.0 (Microcal, USA).

## Results

### Effect of BR Treatment on CI Index of Tomato Fruit

**Figure 1:**
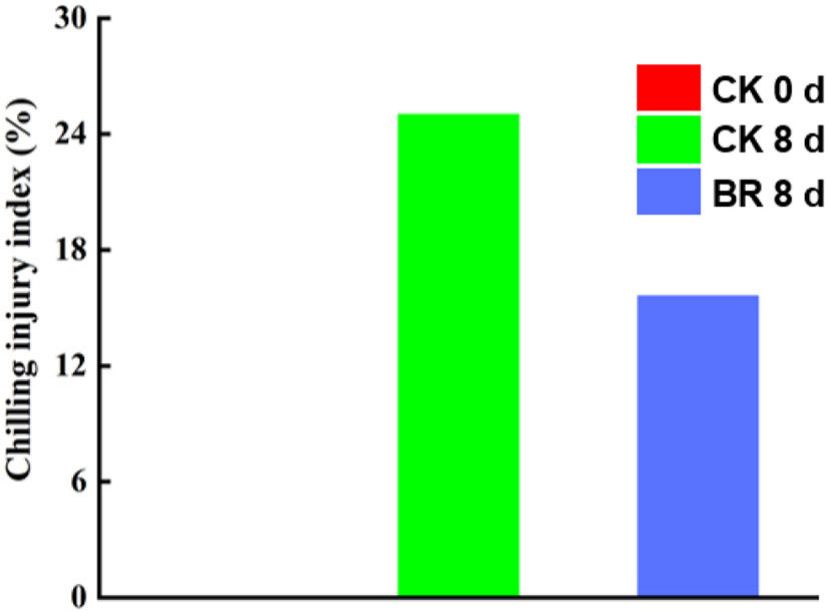
Chilling injury (CI) index of tomato fruit.

After 8 days of storage at 0°C, tomato fruit in both the BR-treated group and the CK group showed symptoms of CI, while the CI index of the fruits in the BR-treated group was 15.6% lower than that of the CK group. The CI indices of the CK group and BR-treated group were 25 and 9.4%, respectively (Figure 1).

### Metabolome Sequencing Analysis of CI in Tomato Fruit During Cold Storage

#### Analysis of Metabolites Differentially Expressed in Response to Chilling in Tomato Fruit

**Figure 2:**
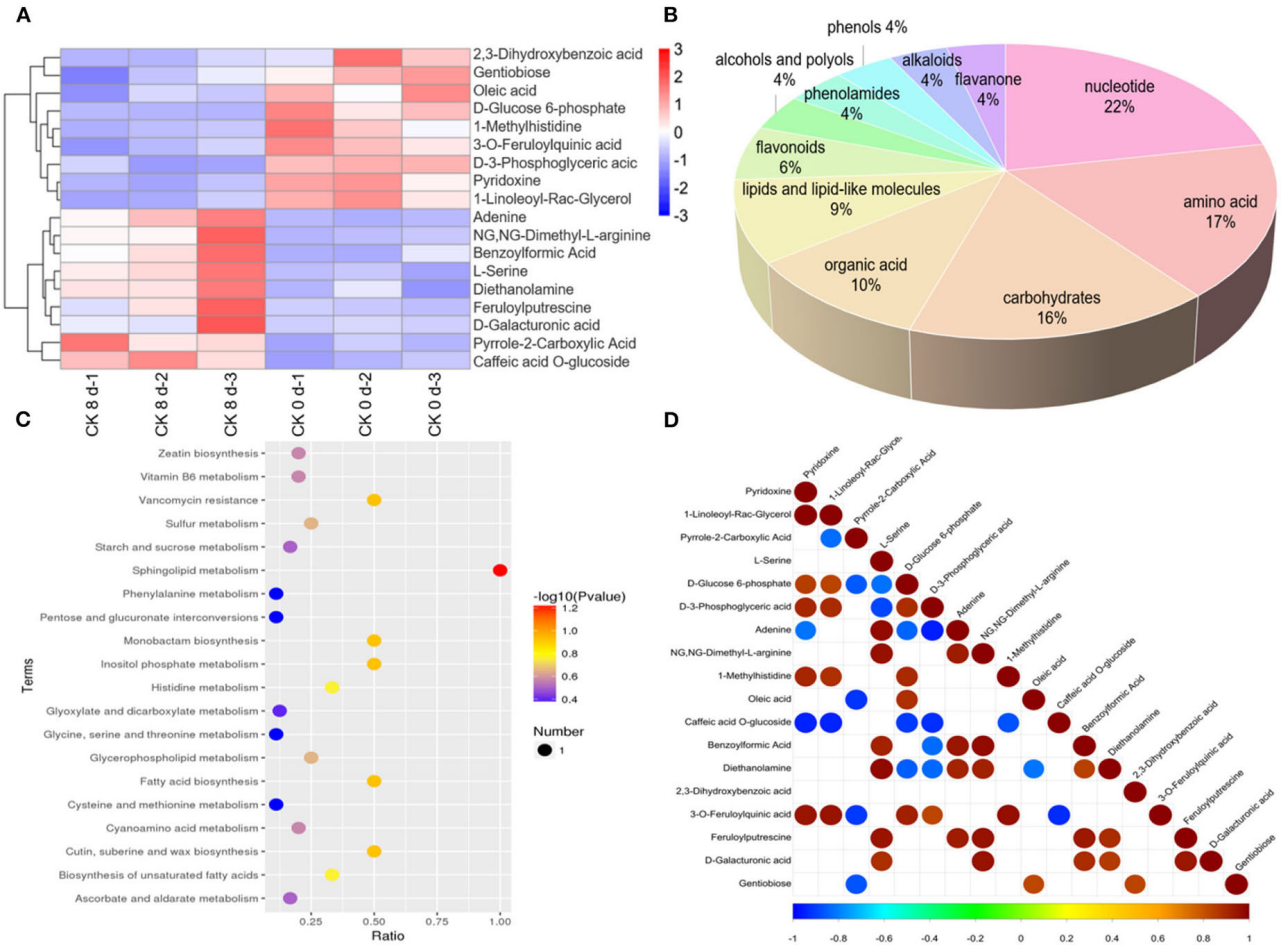
Overview of differentially expressed metabolites. **(A)** Cluster analysis of differentially expressed metabolites s of CK 8- and 0-day group. **(B)** Classification of differential metabolites and their proportion in total differential metabolites of CK 8 d and 0 d groups. **(C)** KEGG pathway analysis of differential metabolites of CK 8- and 0-day group. **(D)** Correlation analysis of differential metabolites of CK 8- and 0-day group.

For CK 8 vs. 0 day, a total of 121 differential metabolites were found to be affected by CI, with 63 downregulated and 58 upregulated. A cluster analysis of the differentially expressed metabolites s revealed different expression patterns between CK 8 and 0 day (Figure 2A). These differentially accumulated metabolites represented the main components in the ripening tomato fruit and were divided into 11 categories, the largest being nucleotides, and amino acids and their derivatives (~22 and 17%, respectively), followed by carbohydrates (16%), organic acid and its derivatives (10%), lipids and lipid-like molecules (9%), flavonoids (6%), alcohols and polyols (4%), phenolamides (4%), phenols (4%), alkaloids (4%), and flavanone (4%) (Figure 2B). KEGG pathway analysis showed that the main changes in response to CI involved metabolites such as sphingolipid and inositol phosphate metabolism, cutin and suberine wax, fatty acid, monobactam biosynthesis, and vancomycin resistance (Figure 2C, Appendix 4).

Through detailed analysis, many differential metabolites were found that were regulated to different degrees during CI. For instance, erucic acid, 1-linoleoyl-rac-glycerol, sarsasapogenin, and D-3-phosphoglyceric acid were downregulated, while notoginsenoside R2 was upregulated in lipids and lipid-like molecules. Moreover, 2, 3-dihydroxybenzoic acid, 2-hydroxyisocaproic acid, lactic acid, and phosphoric acid were downregulated, while pyrrole-2-carboxylic acid was upregulated, in organic acid and its derivatives. Additionally, inositol and D-tagatose were downregulated, while D-galacturonic acid, beta-D-lactose, N-acetyl-D-glucosamine, and sucrose were upregulated, in carbohydrates. Furthermore, tiliroside, lonicerin, and luteolin 7-O-glucoside were greatly downregulated, while methylophiopogonanone A was upregulated, and in flavonoids. Finally, inosine diphosphate and uridine 5′-diphosphate were downregulated, while uracil, cytidine, adenine, adenosine, and uridine were upregulated in nucleotides and their derivatives (Table 1, Appendix 1). In order to determine correlations between the differential metabolites, a correlation analysis was performed and a relationship between the different metabolites was found (Figure 2D).

**Table 1:** Regulation and change fold of differentially expressed metabolites involved in chilling injury (CI) process of CK 8 vs. 0 day group.

**Metabolites**	**Class**	**Fold change**	**Regulation**
1-Linoleoyl-Rac-Glycerol	Lipids and lipid-like molecules	−3.63	DOWN
2,3-Dihydroxybenzoic acid	Organic acid and its derivatives	−4.86	DOWN
2-Hydroxyisocaproic Acid	Organic acid and its derivatives	−2.01	DOWN
Adenine	Nucleotide and its derivates	2.19	UP
Adenosine	Nucleotide and its derivates	2.03	UP
beta-D-Lactose	Carbohydrates	2.49	Up
Cytidine	Nucleotide and its derivates	2.23	Up
D-3-Phosphoglyceric acid	Lipids and lipid-like molecules	−1.95	Down
D-Galacturonic acid	Carbohydrates	16.36	Up
D-Tagatose	Carbohydrates	−1.95	Down
Erucic acid	Lipids and lipid-like molecules	−4.87	Down
Inosine diphosphate	Nucleotide and its derivates	−2.39	Down
Inositol	Carbohydrates	−2.46	Down
Lactic acid	Organic acid and its derivatives	−1.84	Down
Lonicerin	Flavonoids	−7.76	Down
Luteolin 7-O-glucoside	Flavonoids	−4.10	Down
Methylophiopogonanone A	Flavonoids	2.52	Up
N-Acetyl-D-Glucosamine	Carbohydrates	2.46	Up
Notoginsenoside R2	Lipids and lipid-like molecules	1.97	Up
Phosphoric Acid	Organic acid and its derivatives	−1.79	Down
Pyrrole-2-Carboxylic Acid	Organic acid and its derivatives	3.37	Up
Sarsasapogenin	Lipids and lipid-like molecules	−2.67	Down
Sucrose	Carbohydrates	1.83	Up
Tiliroside	Flavonoids	−12.04	Down
Uracil	Nucleotide and its derivates	2.32	Up
Uridine	Nucleotide and its derivates	2.02	Up
Uridine 5′-diphosphate	Nucleotide and its derivates	−2.02	Down

#### Effects of BR on the Differential Accumulation of Metabolites in Chilled Tomato Fruit

**Figure 3:**
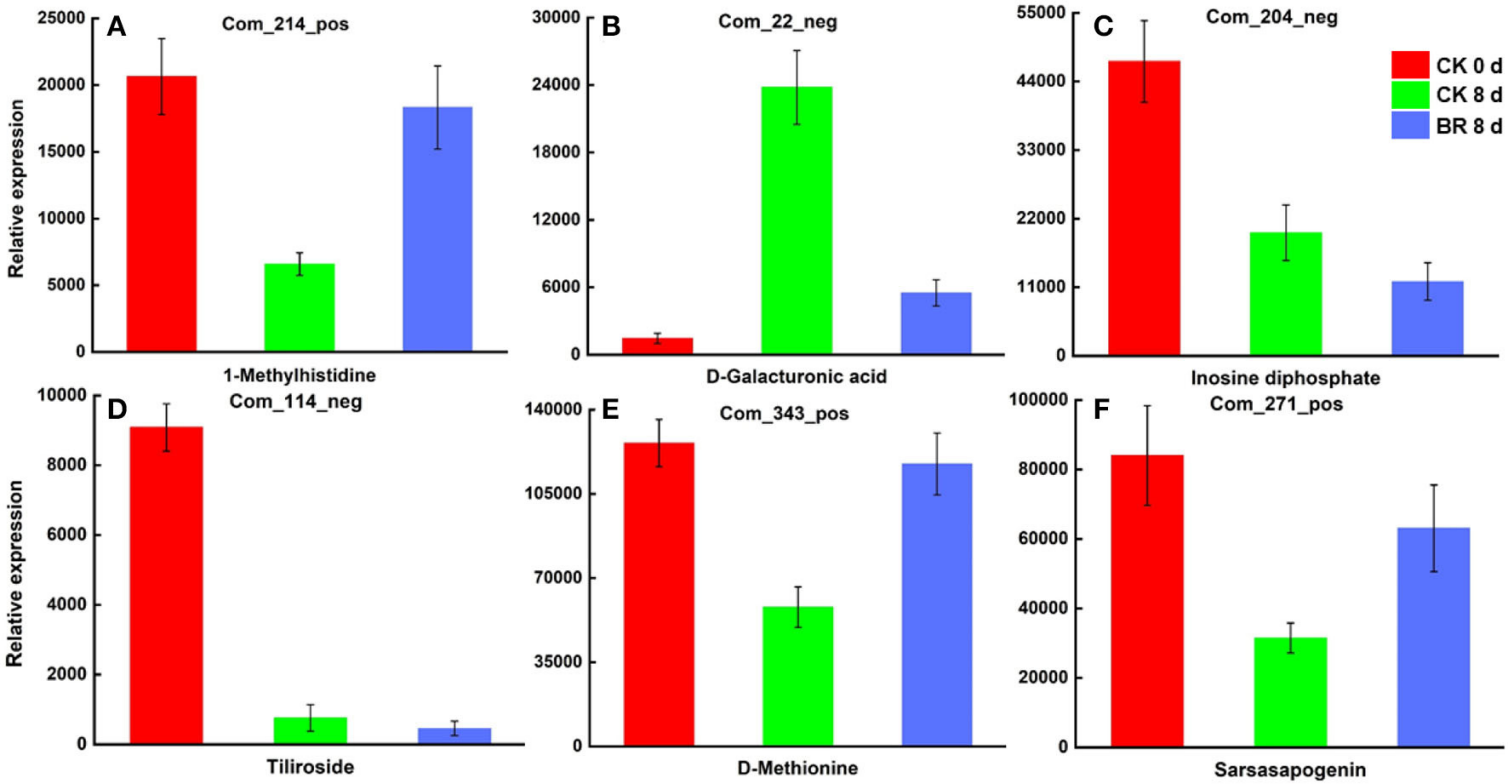
Differentially expressed metabolites in tomato fruit CI process regulated by BR. **(A)** Relative expression of differentially expressed metabolites 1-Methylhistidine. **(B)** D-Galacturonic acid. **(C)** Inosine diphosphate. **(D)** Tiliroside. **(E)** D-Methionine. **(F)** Sarsasapogenin.

The joint analysis results for the two comparison groups (CK 8 vs. 0 day and BR 8 vs. CK 8 days) indicated that 43 differential accumulations of metabolites were regulated by BR to different degrees during the process of CI in tomato fruit. These included homocysteine, 1-methylhistidine, D-methionine and N, N-dimethylglycine in amino acid and its derivatives; uridine 5′-diphosphate, inosine diphosphate, and l-dihydroorotic acid in nucleotides and its derivates; tiliroside and luteolin 7-o-glucoside in flavonoids; D-galacturonic acid in carbohydrates; pyridoxine in vitamins; 2-hydroxyIsocaproic acid, 2,3-dihydroxybenzoic acid, 3-hydroxy-3-methyl butyric acid and lactic acid in organic acid and its derivatives; notoginsenoside R2, erucic acid, 1-linoleoyl-rac-glycerol, sarsasapogenin, and aucubin in lipids and lipid-like molecules (Figure 3).

### Transcriptome Sequencing Analysis of CI in Tomato Fruit During Cold Storage

#### Regulation of DEGs in Tomato Fruit CI

**Figure 4:**
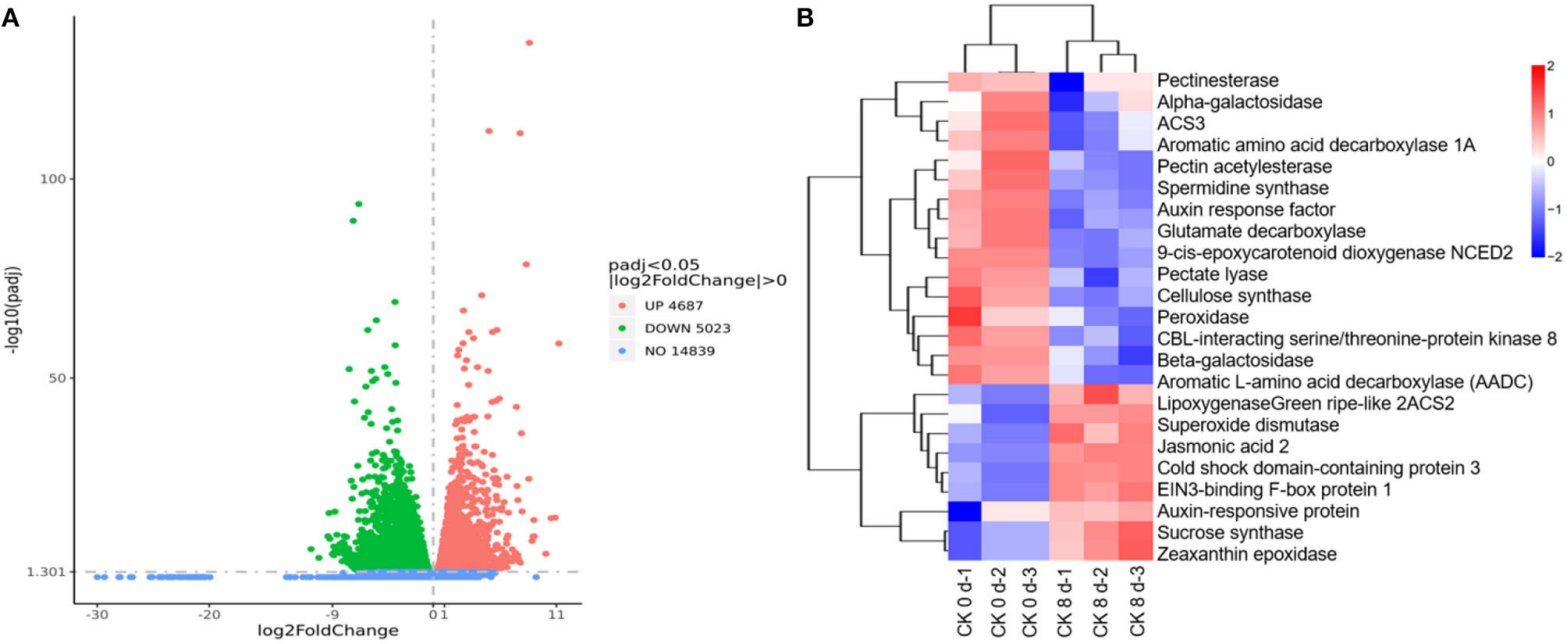
Overview of transcriptome regulation of CI process in tomato fruit. **(A)** The volcanic map of DEGs of CK 8 vs. 0 day group. **(B)** Cluster heat map of DEGs of CK 8-day vs. CK 0-day group.

A total of 9,710 DEGs were found in the CK 8 vs. 0 day group, among which 4,687 were up-regulated, representing 48.27%, and 5,023 (51.73%) were downregulated (Figure 4A). A cluster analysis of the DEGs revealed different expression patterns between CK 8 and 0 day (Figure 4B). The top GO results showed that DEGs are involved in the biological processes including response to heat, cellular response to stress, the cellular macromolecule catabolic process, the cellular protein catabolic process, the cellular amino acid metabolic process, and the cell wall pectin metabolic process. The molecular functions of DEGs mainly included oxidoreductase, beta-galactosidase, and protein kinase regulator activity (Appendix 5). The DEGs involved in pathways such as fatty acid metabolism, fatty acid biosynthesis, and starch and sucrose metabolism were found by KEGG analysis (Appendix 5).

Through careful screening and analysis, a total of 42 DEGs related to CI, of which 24 were downregulated and 18 were upregulated, were found that participated in plant hormone signal transduction. These included *1-aminocyclopropane-1-carboxylate synthase 2 (ACS2), 1-aminocyclopropane-1-carboxylate synthase 3 (ACS3), auxin response factor (ARF), auxin-responsive protein (AUX/IAA), EIN3-binding F-box protein 1 (EBF1), gibberellin 20-oxidase-3 (GA20ox3)*, and *jasmonic acid 2 (JA2)*; genes involved in membrane lipids and cell wall modification, such as *diacylglycerol kinase (DGK), phosphoinositide phospholipase C (PLC), phospholipase (PLA)*, and *phospholipase A(1) LCAT3*; genes involved in biological oxidation processes, including *glutathione S-transferase (GST), lipoxygenase (LOX), peroxidase (POD), serine/threonine-protein kinase (STK)*, and *superoxide dismutase (SOD)*; genes involved in cold stress responses in plants, such as *CBL-interacting serine/threonine-protein kinase 8 (CIPK8), cold shock domain-containing protein 3 (CSP3), stress-associated protein 2 (SAP2)*, and *stress-associated protein 5 (SAP5)*; genes associated with fruit quality, including *aromatic amino acid decarboxylase 1A (AADC1A), aromatic L-amino acid decarboxylase (AADC), glutamate decarboxylase (GAD), glycosyltransferase (GTF), malic enzyme (ME), 9-cis-epoxycarotenoid dioxygenase (NCED2), green ripe-like 2, zeaxanthin epoxidase (ZEP), beta-galactosidase (*β*-GAL), calcineurin B-like molecule (CBL), cellulose synthase (CESA), pectate lyase (PL), pectin acetylesterase (PAE)*, and *pectinesterase (PE)*. Furthermore, a large number of transcription factors (TFs) involved in the chilling response process were identified, including *AP2, bHLH, bZIP, GATA, MYB12, TCP*, and *WRKY* (Table 2, Appendix 2).

**Table 2:** Regulation and change fold of DEGs involved in (CI) process of CK 8 vs. 0 day group.

**Genes**	**Fold change**	**Regulation**
*1-aminocyclopropane-1-carboxylate synthase 2 (ACS2)*	12.72	Up
*1-aminocyclopropane-1-carboxylate synthase 3 (ACS3)*	−25.97	Down
*9-cis-epoxycarotenoid dioxygenase (NCED2)*	−10.40	Down
*AP2 transcription factor*	1.01	Up
*Aromatic amino acid decarboxylase 1A (AADC1A)*	−4.22	Down
*Aromatic L-amino acid decarboxylase (AADC)*	−1.66	Down
*Auxin response factor (ARF)*	−15.09	Down
*Auxin-responsive protein (AUX/IAA)*	22.19	Up
*Beta-galactosidase (β-GAL)*	−15.41	Down
*bHLH transcription factor*	−5.18	Down
*bZIP transcription factor*	1.63	Up
*Calcineurin B-like molecule (CBL)*	1.47	Up
*CBL-interacting serine/threonine-protein kinase 8 (CIPK8)*	−1.71	Down
*Cellulose synthase (CESA)*	−11.88	Down
*Cold shock domain-containing protein 3 (CSP3)*	2.37	Up
*Diacylglycerol kinase (DGK)*	−1.40	Down
*EIN3-binding F-box protein 1 (EBF1)*	6.10	Up
*GATA transcription factor*	−6.13	Down
*Gibberellin 20-oxidase-3 (GA20ox3)*	−10.22	Down
*Glutamate decarboxylase (GAD)*	−12.33	Down
*Glutathione S-transferase (GST)*	1.31	Up
*Glycosyltransferase (GTF)*	5.56	Up
*Green ripe-like 2*	2.01	Up
*Jasmonic acid 2 (JA2)*	6.47	Up
*Lipoxygenase (LOX)*	−1.85	Down
*Malic enzyme (ME)*	−1.84	Down
*MYB transcription factor*	−3.29	Down
*Pectate lyase (PL)*	−181.02	Down
*Pectin acetylesterase (PAE)*	−2.98	Down
*Pectinesterase (PE)*	−20.63	Down
*Peroxidase (POD)*	−54.00	Down
*Phosphoinositide phospholipase C (PLC)*	−2.09	Down
*Phospholipase (PLA)*	0.80	Up
*Phospholipase A (1) LCAT3*	−2.17	Down
*Serine/threonine-protein kinase (STK)*	−1.53	Down
*Stress-associated protein 2 (SAP2)*	1.08	Up
*Stress-associated protein 5 (SAP5)*	1.16	Up
*Sucrose synthase (SUS)*	4.45	Up
*Superoxide dismutase (SOD)*	2.21	Up
*TCP transcription factor*	−3.30	Down
*WRKY transcription factor*	2.37	Up
*Zeaxanthin epoxidase (ZEP)*	2.98	Up

#### Regulation of DEGs in BR in Tomato Fruit CI

**Figure 5:**
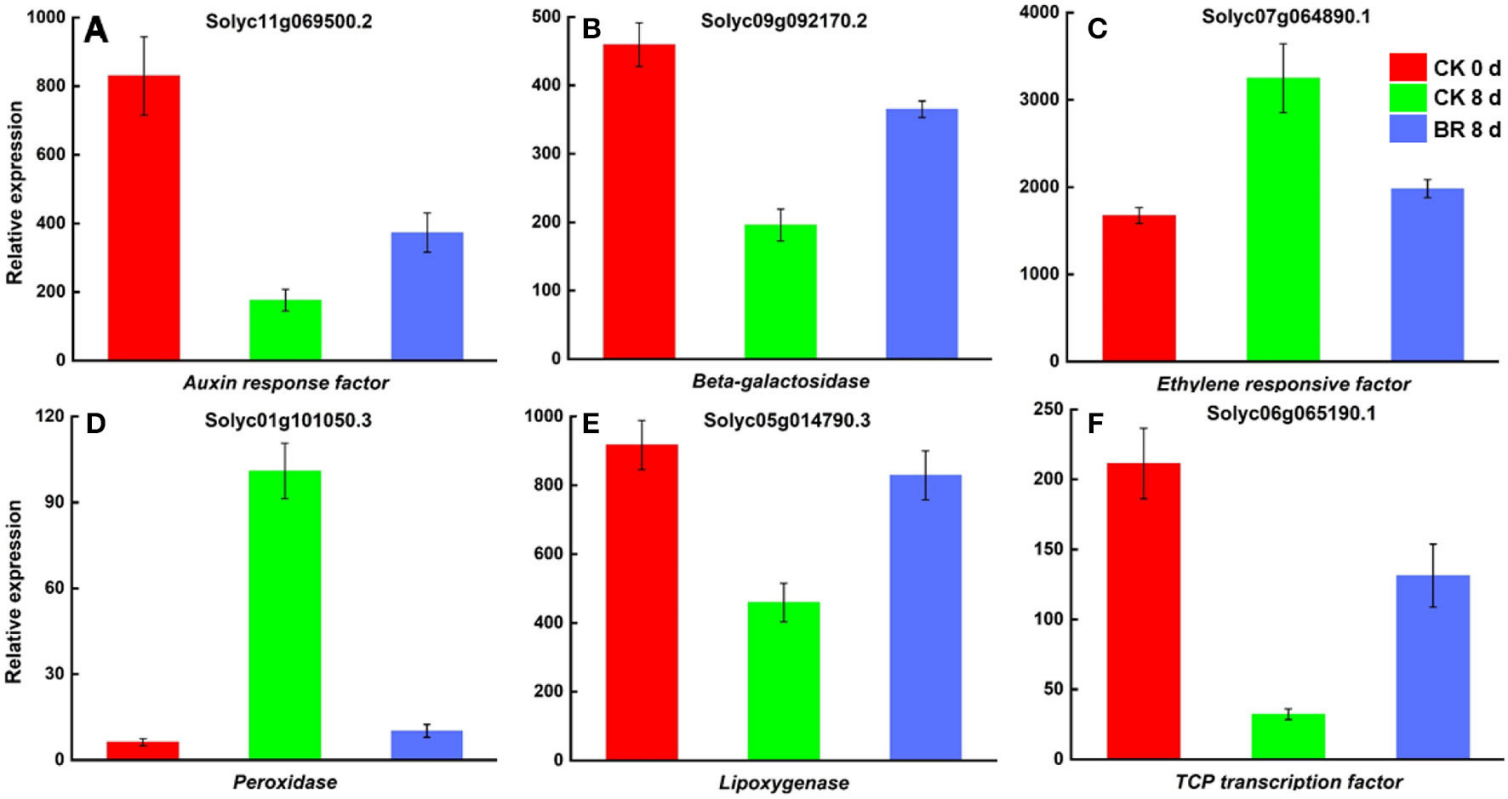
DEGs in tomato fruit CI process regulated by BR. **(A)** Relative expression of mRNA encoding *Auxin response factor (ARF)*. **(B)** *Beta-galactosidase (*β*-GAL)*. **(C)** *Ethylene responsive factor (ERF)*. **(D)** *Peroxidase (POD)*. **(E)** *Lipoxygenase (LOX)*. **(F)** *TCP transcription factor 13*.

The further screening and analysis of the two comparison groups (CK 8 vs. 0 day and BR 8 days vs. CK 0 day) revealed that numerous important genes related to the process of CI in tomato fruit were affected by BR regulation. The DEGs that were discovered involved genes that plant cold stress, including *expansin (EXP)*, the *heat shock protein Dnaj with tetratricopeptide repeat-containing protein (TPR), heat stress transcription factor A3 (HSFA3), MADS-box protein 5, small heat shock protein (SHSP)* and *zinc finger protein constans-like 5*; *peroxidase (POD)*, and *phenylalanine ammonia-lyase (PAL)*, is related to the redox process of tomato fruit CI. Many enzymes were found that were regulated by BR and had effects on the flavor and texture of tomato fruit, including *beta-galactosidase (*β*-GAL), cellulose synthase (CESA), cysteine synthase (CYS), pectin acetylesterase (PAE), sucrose synthase (SUS), xyloglucan endotransglucosylase/hydrolase (XTH), zeaxanthin epoxidase (ZEP), lipoxygenase (LOX)*, and *putative alcohol dehydrogenase (ADH)*. Some DEGs involved in plant hormone and signal transduction were found to be regulated by BR during the CI process, such as *1-aminocyclopropane-1-carboxylate synthase 3 (ACS3), ABC transporter B family member 1(ABCB1), auxin response factor (ARF), auxin transport protein BIG, auxin-regulated protein, EIN3-Binding F-box protein 1(EBF1), ethylene receptor (ETR)*, and *ethylene-responsive factor (ERF)*. Several transcription factors, including *GATA, MADS, TCP, bHLH3*, and *guided entry of tail-anchored proteins factor 4 (GTE4)*, were found to be involved in the regulation of BR in the process of CI in tomato fruit, and *brassinolide LRR receptor kinase (BRI)* was also found to be involved in the regulatory process of CI (Figure 5). Interestingly, it was discovered that BR-regulated DEGs were involved in plant hormone signal transduction, mitogen-activated protein kinase (MAPK) signaling pathways, the biosynthesis of amino acids, phenylpropanoid biosynthesis, carotenoid biosynthesis, and starch and sucrose metabolism during CI.

#### Combined Transcriptome and Metabolome Analysis

**Figure 6:**
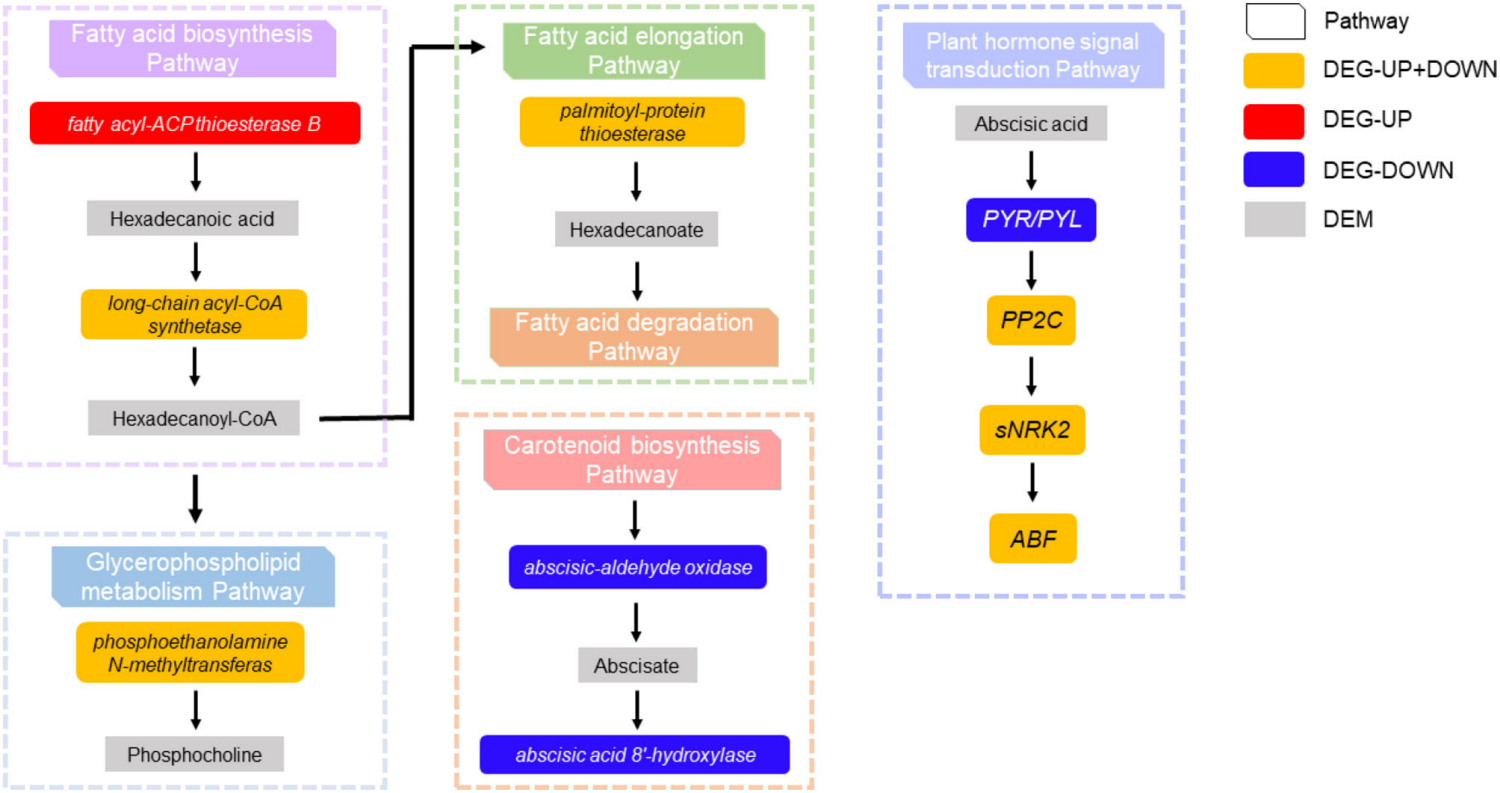
The interaction between DEGs and differentially expressed metabolites were found in the fatty acid biosynthesis pathway, glycerophospholipid metabolism pathway, fatty acid elongation pathway, carotenoid biosynthesis pathway, and plant hormone signal transduction pathway.

In the fatty acid biosynthesis pathway, it was found that the expression of palmitoyl-acyl carrier protein thioesterase in tomato fruit affected by CI was upregulated, resulting in the annotation of the metabolite hexadecanoic acid, affecting the accumulation of *long chain-Acyl-CoA synthetase 2*. In turn, phosphoethanolamine N-methyltransferase in the glycerophospholipid metabolism pathway was regulated to varying degrees, affecting the accumulation of choline phosphate. Additionally, changes in the expression of *palmitoyl-protein thioesterase* in fatty acid elongation led to the annotation of hexadecanoate, which affects the fatty acid degradation pathway. It was also found that *short-chain dehydrogenase-reductase (SDR)* was downregulated in carotenoid biosynthesis, which affected the accumulation of abscisic aldehyde, resulting in abscisic acid 8'-hydroxylase being downregulated. The regulation of *PYR/PYL, PP2C*, and *ABF* by abscisic acid in the plant hormone signal transduction pathway was affected. Finally, the interaction between the DNA and stomatal closure seed dormancy was terminated (Figure 6).

### Proteome Sequencing Analysis of CI in Tomato Fruit During Cold Storage

#### Differential Protein Analysis in Tomato Fruit CI

The proteome was quantitatively analyzed to determine protein amounts in fruit with and without CI. A total of 7,865 proteins were identified in the tomato fruit protein samples. Among the 7,865 proteins, 308 were found to be differentially expressed between CI and non-CI, of which 153 were downregulated and 155 were upregulated. The KEGG pathway enrichment analysis showed that the differentially expressed proteins were majorly enriched in four pathways: phagosomes, fatty acid biosynthesis, phenylalanine metabolism, and amino sugar and nucleotide sugar metabolism (Appendix 6). A GO functional enrichment analysis was performed to elucidate the molecular functions and biological processes involved in differentially expressed proteins between chilling and non-chilling tomato fruit (Appendix 6). According to the enrichment degrees, differentially expressed proteins were mainly involved in six groups of CI biological processes, including the cellular protein modification process (15 proteins), response to stimulus (11 proteins), the carbohydrate metabolic process (10 proteins), the organic acid metabolic process (8 proteins), the cellular amino acid metabolic process (5 proteins), and the alpha-amino acid metabolic process (4 proteins).

After careful screening and analysis, the differentially expressed proteins related to the CI process were found. Among these were differentially expressed proteins associated with fruit texture such as beta-galactosidase (β-GAL), beta-glucanase (BGL), calcium-binding protein (CML), cellulase/cellobiase (CelA1), cellulose synthase A(CESA), chitinase (CHI), glycosyltransferase (GTF), and pectinesterase (PE); differentially expressed proteins associated with fruit flavor and aroma, including fatty acid omega-hydroxylase (FAωH), glutamate decarboxylase (GAD), glutamate-1-semialdehyde 2,1-aminomutase (GSAM), glutamine synthetase (GS), IAA-amino acid hydrolase (ILL), and polygalacturonase (PGL); differentially expressed proteins involved in plant oxidation processes, including glutathione S-transferase (GST), predicted ATPase and serine/threonine-protein kinase (STK), heat shock transcription factor (HSF) involved in plant cold stress response, and WRKY transcription factor 33 (Table 3, Appendix 3).

**Table 3:** Regulation and change fold of differentially expressed proteins involved in CI process of CK 8- vs. 0-day group.

**Proteins**	**Change fold**	**Regulation**
Fatty acid omega-hydroxylase (FAωH)	−1.43	Down
Glutamate decarboxylase (GAD)	−1.49	Down
Glutamate-1-semialdehyde 2,1-aminomutase (GSAM)	2.91	Up
Glutamine synthetase (GS)	−2.19	Down
IAA-amino acid hydrolase (ILL)	1.73	Up
Polygalacturonase (PGL)	1.95	Up
Heat shock transcription factor (HSF)	−1.69	Down
Glutathione S-transferase (GST)	−1.86	Down
Predicted ATPase	1.83	Up
Serine/threonine-protein kinase (STK)	−1.81	Down
Beta-galactosidase (β-GAL)	−2.19	Down
Beta-glucanase (BGL)	2.05	Up
Calcium-binding protein (CML)	−2.13	Down
Cellulase/cellobiase (CelA1)	3.12	Up
Cellulose synthase A(CESA)	3.13	Up
Chitinase (CHI)	−1.59	Down
Glycosyltransferase (GTF)	2.21	Up
Pectinesterase (PE)	−1.44	Down
WRKY transcription factor 33	−1.75	Down

#### Differential Protein Analysis of BR in Tomato Fruit CI

**Figure 7:**
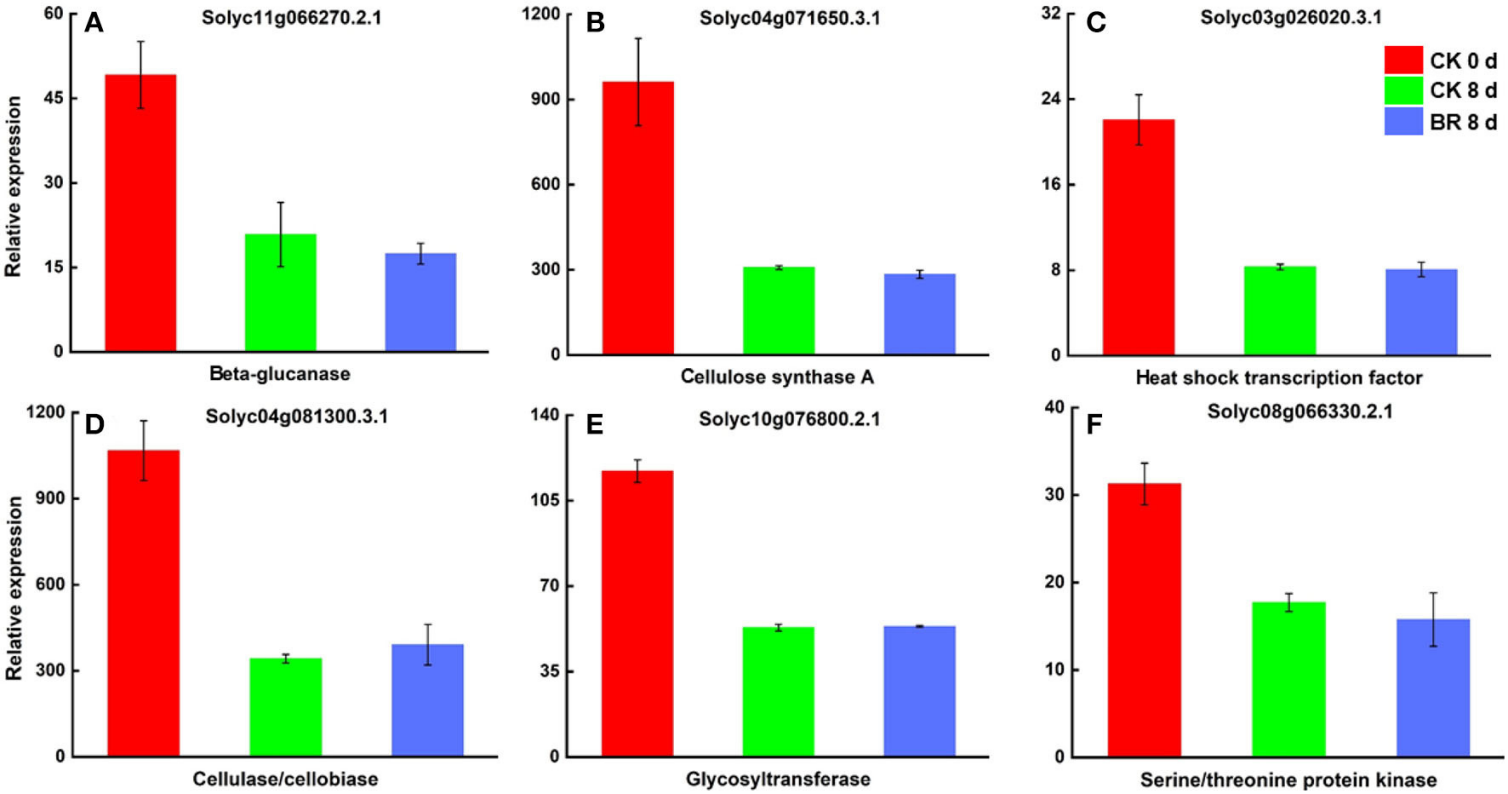
Differentially expressed proteins in tomato fruit CI process regulated by BR. **(A)** Relative expression of beta-glucanase (BGL). **(B)** Cellulose synthase A (CESA). **(C)** Heat shock transcription factor (HSF). **(D)** Cellulase/cellobiase (CelA1). **(E)** Glycosyltransferase (GTF). **(F)** Serine/threonine-protein kinase (STK).

By screening and analyzing the BR 8 vs. CK 8 day group, many different proteins that may be regulated by BR during CI were found. Among these, they were differentially expressed proteins associated with fruit texture, such as cellulose synthase A (CESA), pectate lyase (PL), pectinesterase (PE), chitinase (CHI), and beta-galactosidase (β-GAL); differentially expressed proteins associated with fruit flavor and aroma, including IAA-amino acid hydrolase (ILL), glutamine synthetase (GS), sucrose synthase (SUS), and fatty acid desaturase (FADS); differentially expressed proteins involved in plant oxidation processes including lipoxygenase (LOX), glutathione peroxidase (GPX), serine/threonine kinase 38 (STK38), catalase (CAT), predicted ATPase, serine/threonine protein kinase (STK), peroxidase (POD), and glutathione S-transferase (GST); differentially expressed proteins associated with in plant cold stress response such as heat shock factor-binding protein 1 (HSBP1), heat shock protein 90 kDa beta (HSP90B), heat shock transcription factor (HSF), heat shock 70 kDa protein (HSP70), and HSP20 family protein (HSP20); differentially expressed proteins involved in plant hormone signal transduction such as BR-signaling kinase (BSK1), jasmonic acid-amino synthetase (JAR1) and WRKY and MYB transcription factors (TFs) (Table 4). The two comparison groups were combined (CK 8 vs. 0 day and BR 8 days vs. CK 0 day) to identify six different proteins that were regulated by BR during CI of tomato fruit, which was cellulose synthase A (CESA), beta-glucanase (BGL), heat shock transcription factor (HSF), cellulase/cellobiase (CelA1), glycosyltransferase (GTF), and Serine/threonine-protein kinase (STK) (Figure 7).

**Table 4:** Regulation and change fold of differentially expressed proteins involved in CI process of BR 8 vs. CK 8 day group.

**Proteins**	**Fold change**	**Regulation**
IAA-amino acid hydrolase (ILL)	1.186186186	Up
Glutamine synthetase (GS)	−1.188927541	Down
Sucrose synthase (SUS)	1.273990735	Up
Fatty acid desaturase (FADS)	1.403634255	Up
Heat shock factor-binding protein 1 (HSBP1)	1.067869008	Up
Heat shock protein 90kDa beta (HSP90B)	−1.126144978	Down
Heat shock transcription factor (HSF)	1.133561644	Up
Heat shock 70kDa protein (HSP70)	−1.597787553	Down
HSP20 family protein (HSP20)	−3.719528178	Down
Lipoxygenase (LOX)	−1.177125658	Down
Glutathione peroxidase (GPX)	−1.186231905	Down
Serine/threonine kinase 38 (STK38)	1.19047619	Up
Catalase (CAT)	−1.251008878	Down
Predicted ATPase	−1.256013746	Down
Serine/threonine-protein kinase (STK)	−1.481707317	Down
Peroxidase (POD)	1.594393922	Up
Glutathione S-transferase (GST)	−2.544230267	Down
A BR-signaling kinase (BSK1)	−1.102171137	Down
Jasmonic acid-amino synthetase (JAR1)	−1.105448155	Down
Cellulose synthase A (CESA)	−1.08180007	Down
Pectate lyase (PL)	−1.106422018	Down
Pectinesterase (PE)	1.333048961	Up
Chitinase (CHI)	1.544446625	Up
Beta-galactosidase (β-GAL)	−1.595154185	Down
MYB transcription factor	−1.164571429	Down
WRKY transcription factor 33	−1.204494382	Down

#### Combined Transcriptomic, Metabolites, and Proteomics Analysis

**Figure 8:**
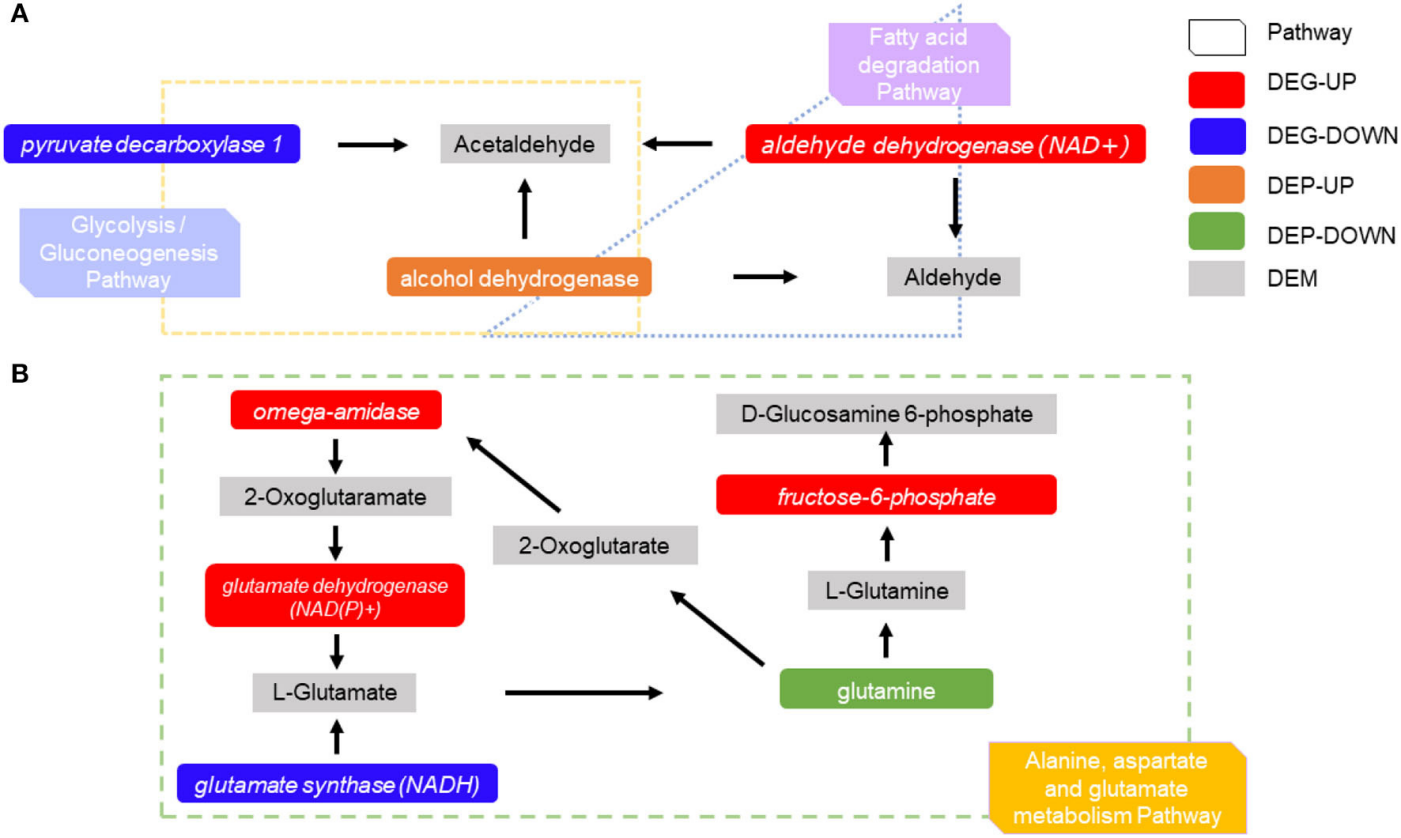
Differentially expressed proteins differentially expressed metabolites, and DEGs are regulated in the pathway. **(A)** The interaction between related DEGs differentially expressed proteins, and differentially expressed metabolites in the fatty acid degradation pathway and glycolysis/gluconeogenesis pathway. **(B)** The interaction between related DEGs has differentially expressed proteins, and differentially expressed metabolites in alanine, aspartate, and glutamate metabolism pathway.

A combination of transcriptomic and proteomic analyses revealed many different substances that were produced by the interaction between genes, metabolites, and proteins. The *aldehyde dehydrogenase (ADH)* of DEGs and the alcohol dehydrogenase 1/7 of differentially expressed proteins appeared in the fatty acid degradation pathway and the glycolysis/gluconeogenesis pathway, and they acted together to produce acetaldehyde and aldehyde (Figure 8A). In alanine, aspartate, and the glutamate metabolism pathway, three DEGs were found to be up-regulated [*omega amidase, glutamate dehydrogenase (GDH), and glutamine-fructose 6-phosphate transaminase (GFPT)*] and *glutamate synthase (GS)* was downregulated, while differentially expressed proteins glutamine synthetase was downregulated, which produced five differentially expressed metabolites (2-oxoglutaramate, L-glutamate, D-glucosamine 6-phosphate, L-glutamine, and 2-oxoglutarate). Meanwhile, 2-oxoglutarate stimulated the upregulation of omega amidase (Figure 8B).

## Discussion

Tomato fruit stored at 0°C is susceptible to CI, which reduces the nutritional quality of the fruit and prevents its normal change of color from affecting the ripening process (39, 40). BR applied to the fruit prior to storage can alleviate CI in several species (green bell pepper, bamboo shoot, rice) of fruit during cold storage (17, 24, 41). In our study, compared with untreated tomato fruit, the BR-treated fruit showed fewer symptoms of the chilling response. These findings suggest that BR is useful for improving the cold tolerance of tomato fruit.

A color change is one of the most important indicators for judging the ripening of tomato fruit (42). Lycopene and β-carotene in the pathway of carotenoids are important components of ripe tomato fruit (43). The degradation of chlorophyll, along with the synthesis of lycopene and β-carotene, causes the fruit to change color from green to orange and eventually to red (44). Studies have shown that *phytoene synthase (PSY)*, encoding a key enzyme in the carotenoid biosynthesis pathway, is induced in expression during the fruit-ripening process (45, 46). Our results showed that *PSY* was also upregulated throughout the storage period in tomato fruit, despite CI, which was consistent with previous reports. Moreover, *PSY* was upregulated to a greater extent in the BR-treated fruit than in the control fruit, indicating that BR could indeed inhibit the CI process of tomato fruit. However, in our results, no significant changes in the genes encoding chlorophyll, lycopene, or beta-carotene were found. When the fruit was transferred from 0°C to room temperature, it was not able to change from green to red normally and accelerated decayed. Therefore, we hypothesized that the genes encoding lycopene and β-carotene in the tomato fruit that was subjected to low-temperature stress were inhibited, resulting in the abnormal color transformation.

β*-Galactosidase* and *pectin methylesterase (PME)* in polysaccharides together form the complex structure of plant cell wall and participates in cell wall modification which is the key enzyme in cell wall degradation (47–51). The synergistic effects of various cell-wall-modification enzymes promote the degradation of cell-wall polysaccharides, among which pectin degradation is the main reason for fruit softening (52). *CEL* is involved in fruit ripening (53). *Endoglucanase (EG)* is one of the most important components of *CEL* due to its ability to degrade the cellulose matrix (50). Studies have found that the activities of *PME* and β*-GAL* increase with the ripening of tomato fruit, along with a decrease in fruit hardness (54–59). However, we found that the genes encoding β*-GAL, EG, pectin lyase (PL)*, and *CESA* were downregulated in tomato fruit is subjected to low-temperature stress and CI. This was not consistent with previous studies and may be caused by the dysfunction of the internal organism of the tomato fruit, which no longer had the ability to ripen normally. However, the genes encoding β*-GAL* and *PL* in BR-treated tomato fruit were upregulated. The results showed that BR can alleviate the symptoms in tomato fruit CI.

**Figure 9:**
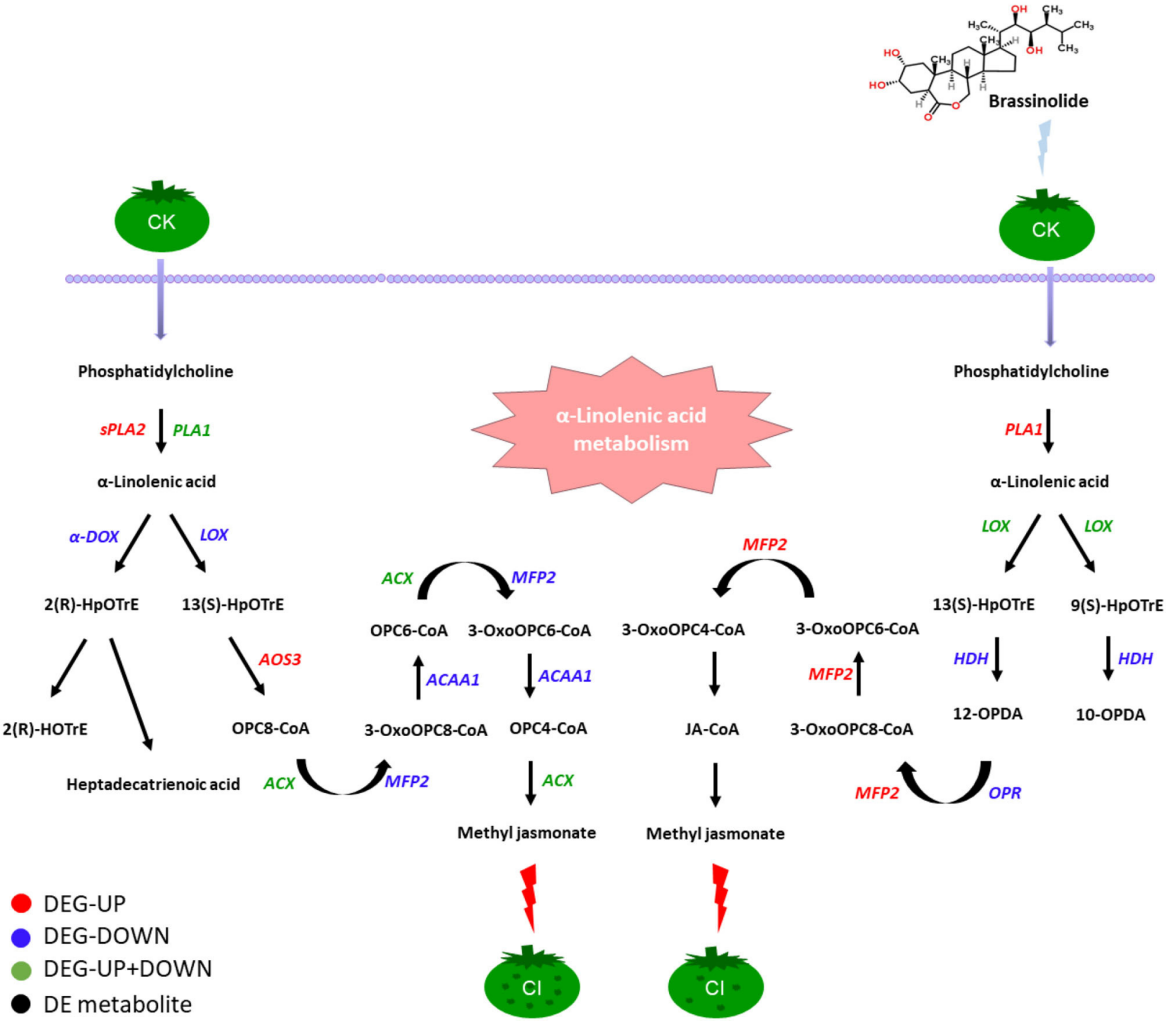
Changes of genes and related enzymes involved in the CI process of tomato fruit under the regulation of BR. Among them, black fonts indicate differentially expressed metabolites, red fonts indicate that DEGs were upregulated, blue fonts indicate that DEGs were downregulated, and green fonts indicate that DEGs are both upregulated and downregulated and black fonts indicate those differential metabolites.

During fruit ripening, the fatty-acid pathway and the amino-acid pathway play major roles in the synthesis of the fruit's aromatic volatiles (60). Studies have shown that the expression of lipid compounds depends on the expression of branched-chain amino acid transaminase (61). Amino-acid metabolism is also thought to be closely related to the cold-resistance mechanism of fruit (62, 63). It is well-known that *LOX, ADH*, and *propylene oxide cyclase* are important enzymes in the fatty-acid metabolism pathway that affect the synthesis of fruit-flavor substances (40). It has been reported that low temperatures can inhibit the activity of *ethanol dehydrogenase (EDH)* (7, 64). Our results were consistent with this; the gene encoding *EDH* was downregulated during CI. Linolenic acid is processed by *9-lipoxygenase (9-LOX)* to form 9-hydrogen peroxide, which, in turn, is processed by *ADH* to produce aromatic volatile substances (65). When linoleic acid is acted upon by *LOX*, the phospholipid bilayer is destroyed, reducing the cold resistance of the fruit (66, 67). In our study, BR increased the expression of the genes encoding *9-LOX* and *EDH*, so the treatment of the fruit with BR was an effective way to maintain the fatty-acid biosynthesis in the formation of aroma substances and effectively alleviate the CI process. Studies have shown that the accumulation of proline is helpful in alleviating CI in fruits, while proline dehydrogenase (ProDH) negatively affects the accumulation of proline (24, 68–70). Our results showed that ProDH was inhibited by BR, which increased the accumulation of proline and increased the cold resistance of the fruit, consistent with previous reports. A putative regulatory network model involving CI in response to BR treatment in the α-linolenic acid metabolism pathway was proposed (Figure 9). In this pathway, phosphatidylcholine can first produce α-linolacid through a series of biosyntheses, and, finally, produce methyl jasmonate through the action of certain enzymes and CoA, relieving the symptoms of CI in tomato fruit.

## Data Availability Statement

The data presented in the study are deposited in the ProteomeXchange Consortium repository, accession number PXD028500.

## Author Contributions

CB and DM wrote the manuscript. QW and JZ offers experimental ideas. YZ analyzed the data. ZY revised the manuscript. AF, LM, HG, SY, SZ, and LG sorted out the pictures and attachments in the manuscript. All authors contributed to the article and approved the submitted version.

## Funding

This work was supported by the National Natural Science Foundation of China (31772022 and 32072284), the Special innovation ability construction fund of Beijing Academy of Agricultural and Forestry Sciences (20210437, 20210402, and 20200427), the Young Investigator Fund of Beijing Academy of Agricultural and Forestry Sciences (202016), the China Agriculture Research System of MOF and MARA (CARS-23), Beijing Municipal Science and Technology Commission (Z191100008619004, Z191100004019010, and Z181100009618033), the Collaborative innovation center of Beijing Academy of Agricultural and Forestry Sciences (201915), Special innovation ability construction fund of Beijing Vegetable Research Center, and Beijing Academy of Agriculture and Forestry Sciences (2020112).

## Conflict of Interest

The authors declare that the research was conducted in the absence of any commercial or financial relationships that could be construed as a potential conflict of interest.

## Publisher's Note

All claims expressed in this article are solely those of the authors and do not necessarily represent those of their affiliated organizations, or those of the publisher, the editors and the reviewers. Any product that may be evaluated in this article, or claim that may be made by its manufacturer, is not guaranteed or endorsed by the publisher.
